# Supplementary material for: Origin of Circumpolar Deep Water intruding onto the Amundsen and Bellingshausen Sea continental shelves
Source: Nat Commun. 2018 Aug 24;9:3403. doi: 10.1038/s41467-018-05813-1 (PMC6109117; doi:10.1038/s41467-018-05813-1)
Supplement: Supplementary file 1 — Supplementary Information [file 41467_2018_5813_MOESM1_ESM.pdf]

Supplementary Information

Origin of Circumpolar Deep Water intruding onto the Amundsen and Bellingshausen Sea continental shelves

Nakayama et al.

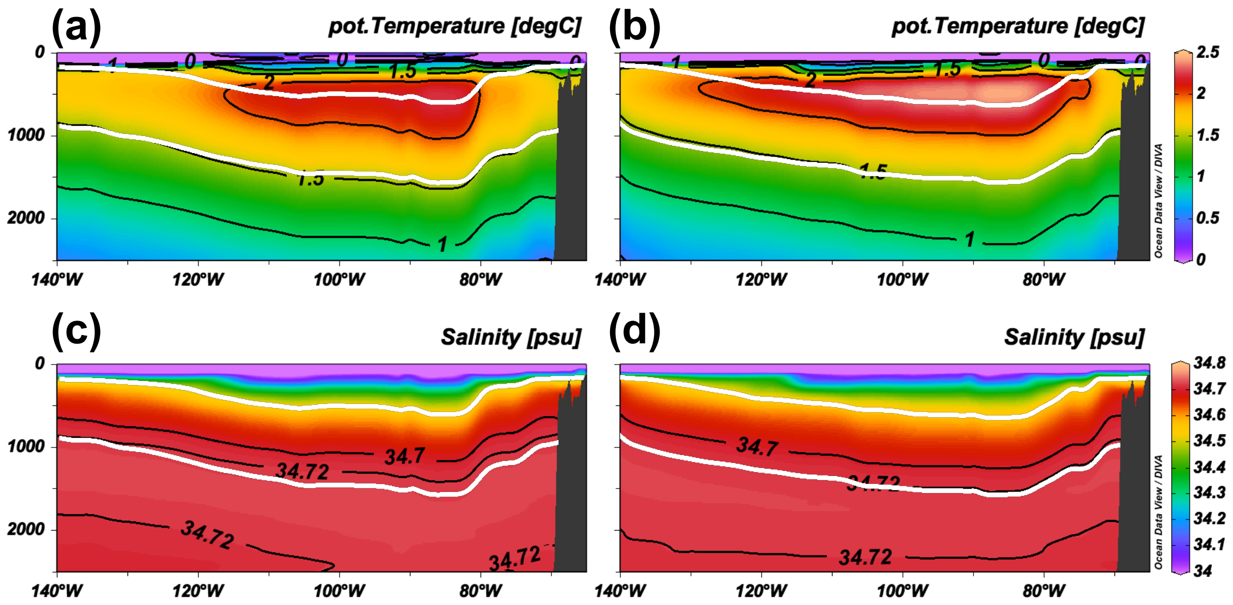

Supplementary Figure 1. Temporally averaged northern boundary conditions between 2001-2006 for (a) potential temperature and (c) salinity. Temporally averaged northern boundary conditions between 2009-2011 for (b) potential temperature and (d) salinity.

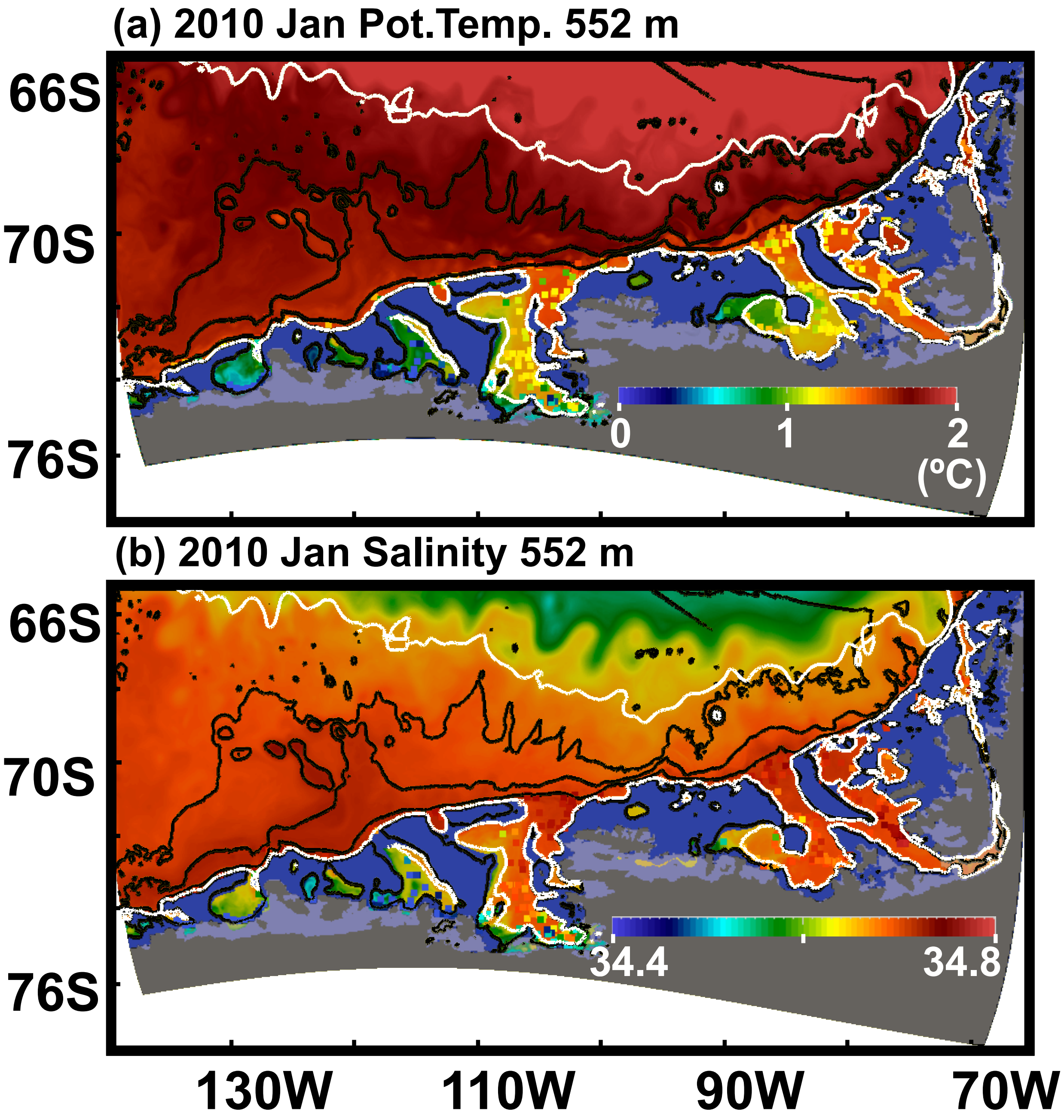

Supplementary Figure 2. Simulated monthly mean (a) potential temperature and (b) salinity at 552-m depth in January 2010. Squares indicate the location of CTD and seal CTD data of 2007, 2009, and 2010 and the color of the squares shows their (a) potential temperature and (b) salinity at 552-m depth similarly to (*16*). Isohalines of 34.65 are shown with white lines.

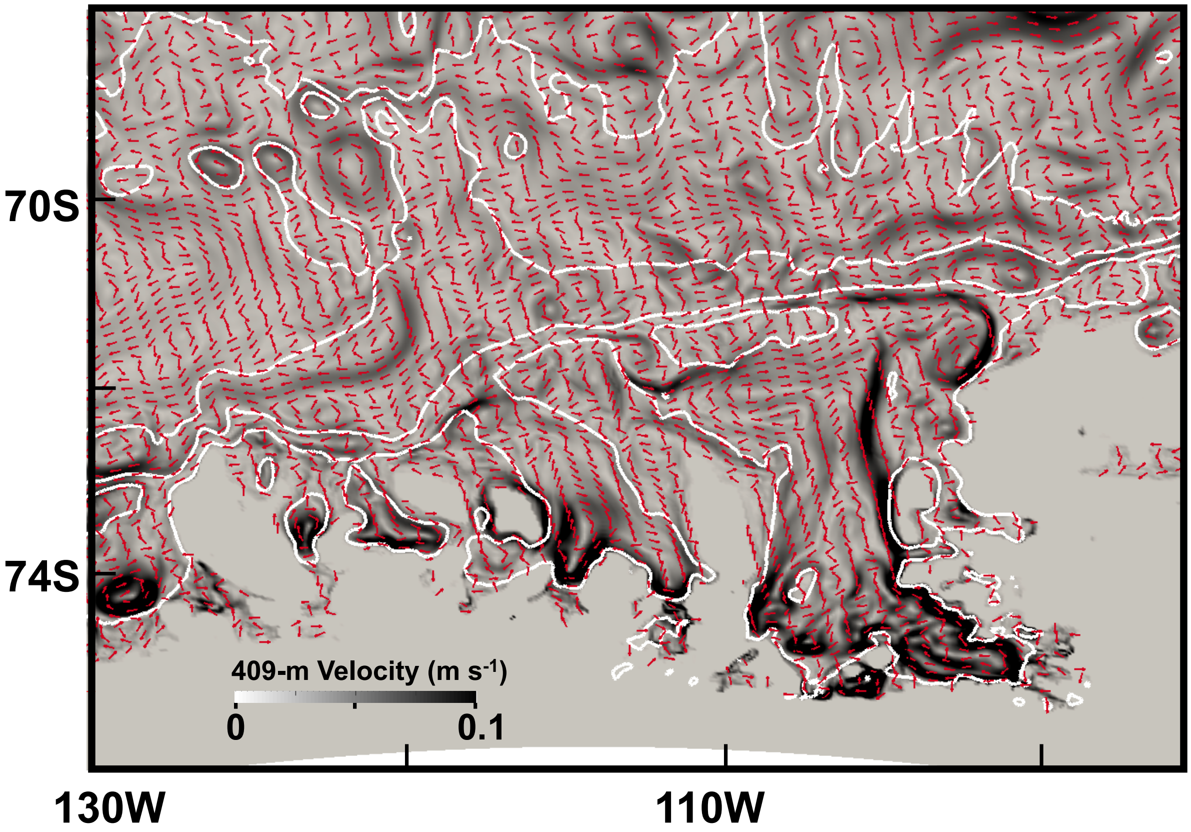

Supplementary Figure 3. Simulated monthly mean velocity (month 15) at the 409-m depth with direction (red arrows) and speed (shade).

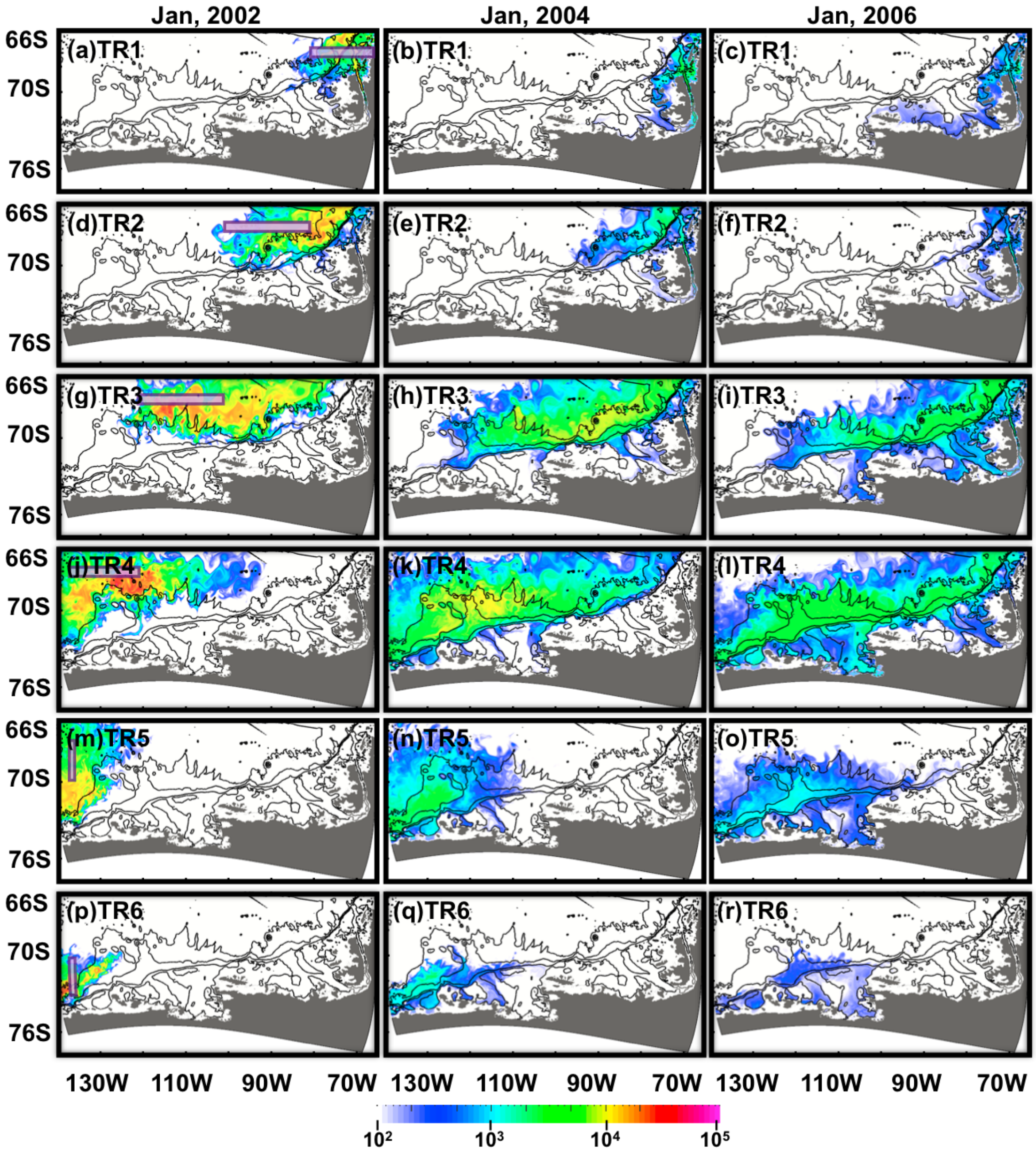

Supplementary Figure 4. Monthly mean spatial distributions of vertically integrated tracers (TR) representing CDW after 1 (Jan 2002), 3 (Jan 2004), and 5 (Jan 2006) years of model simulation. TR (a-c)1, (d-f)2, (g-i)3, (j-l)4, (m-o)5, and (p-r)6 are used to investigate the CDW pathways from different locations and initial locations of these tracers are shown by the purple boxes in the left panels. Close up of the region enclosed by the red rectangle (a) is shown in Fig. 5. For all panels, bathymetric contours of 500, 1500, 3000 and 4000 m are shown with thin black contours.

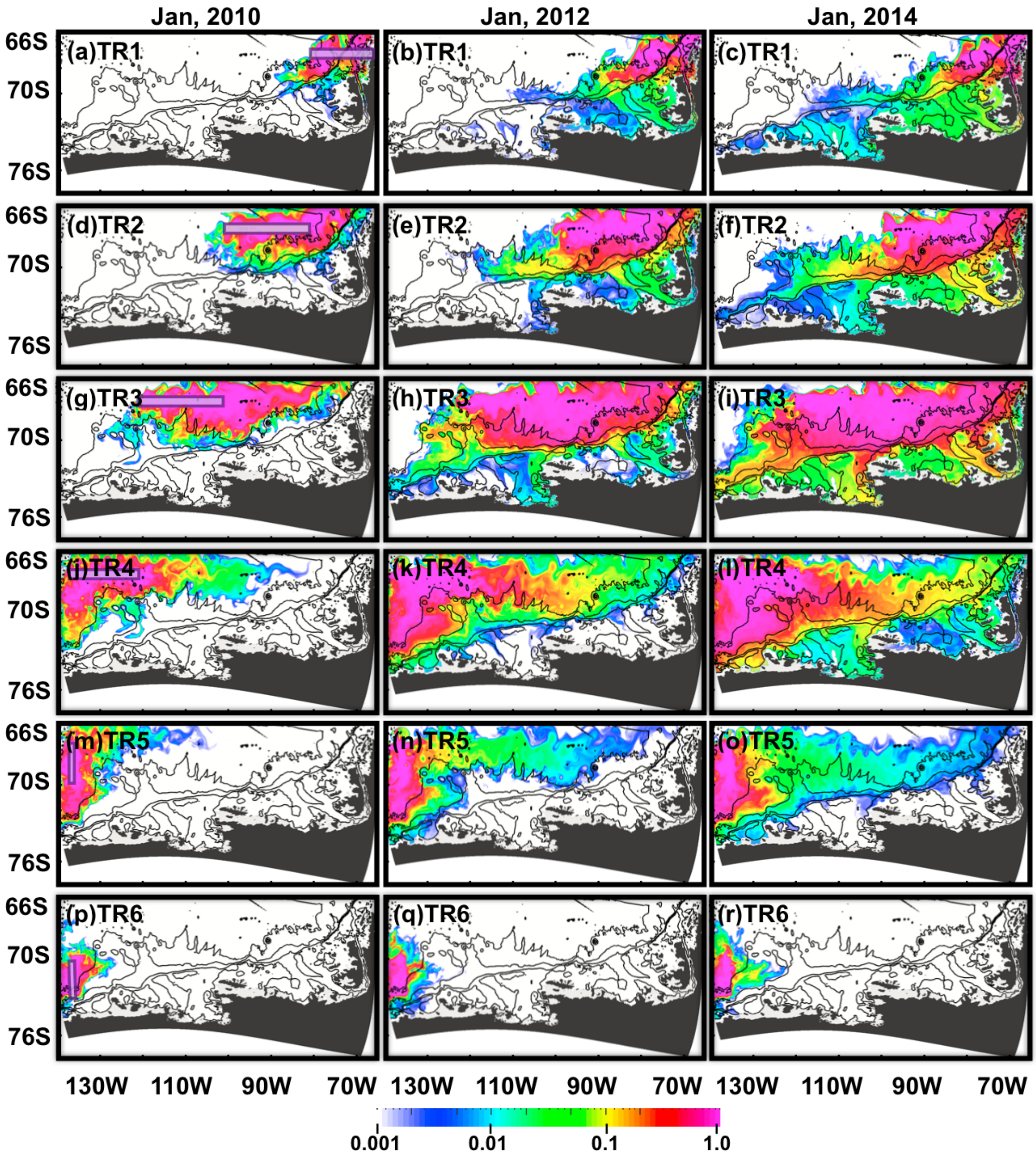

Supplementary Figure 5. Same as Supplementary Figure 4 but for additional sensitivity experiments with restoring tracer for CTRL(2009-2014) case. Note that tracer concentrations are restored to 1.0 in the purple boxes shown in the left panels with a restoring time scale of 1 hour.

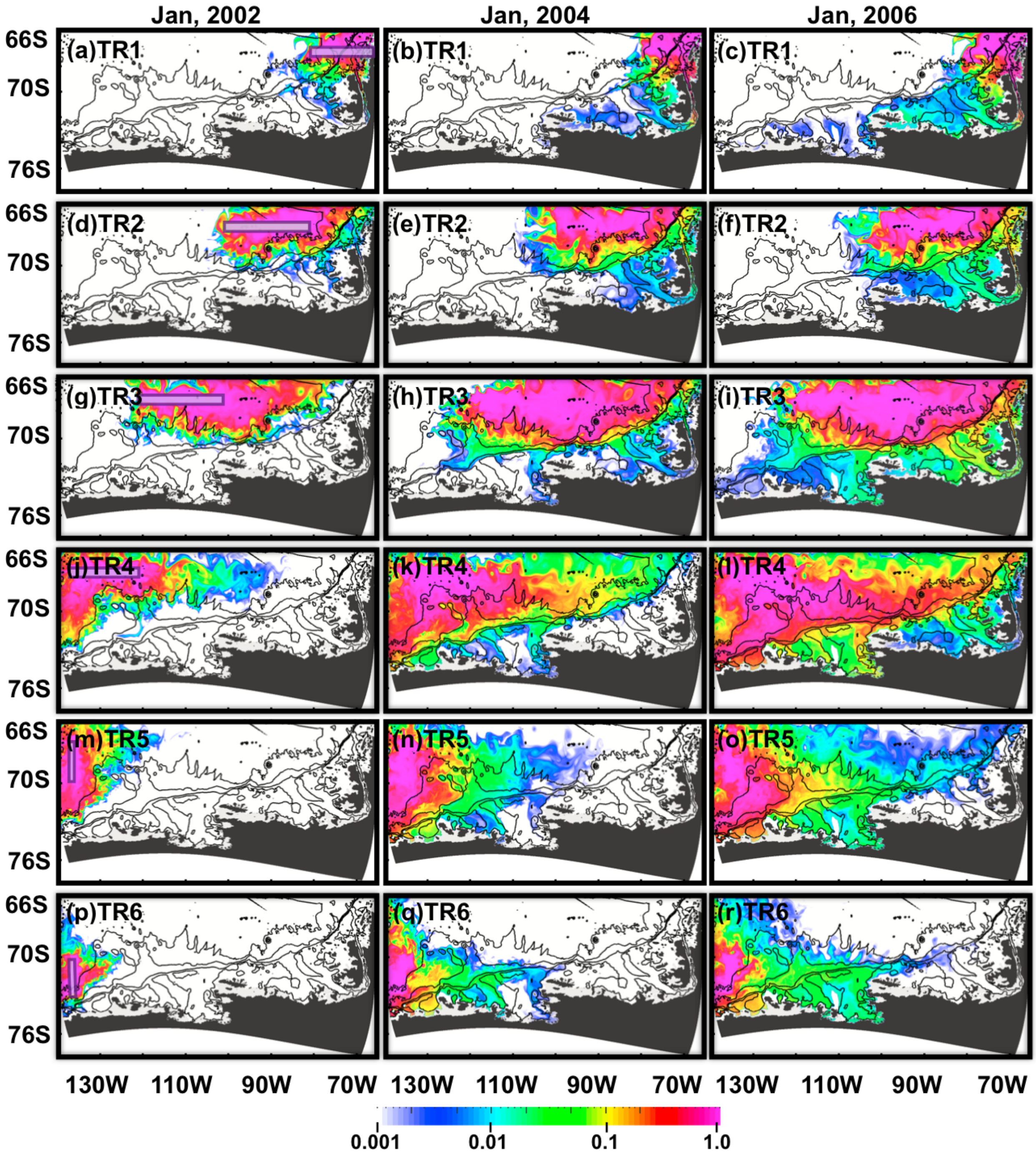

Supplementary Figure 6. Same as Supplementary Figure 4 but for additional sensitivity experiments with restoring tracers for the 2001-2006 case. Note that tracer concentrations are restored to 1.0 in the purple boxes shown in the left panels with a restoring time scale of 1 hour.

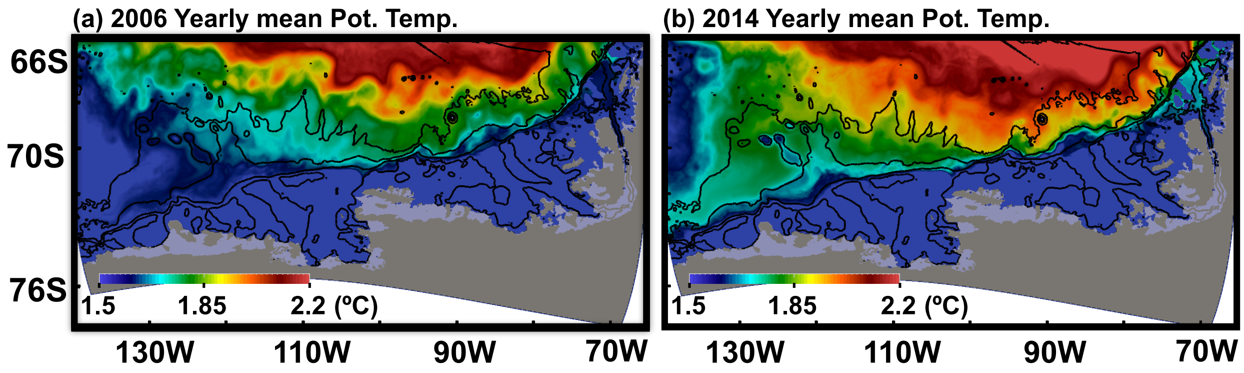

Supplementary Figure 7. Yearly mean 409-m potential temperature for (a) 2006 and (d) 2014 for the 2001-2006 and CTRL(2009-2014) cases, respectively.

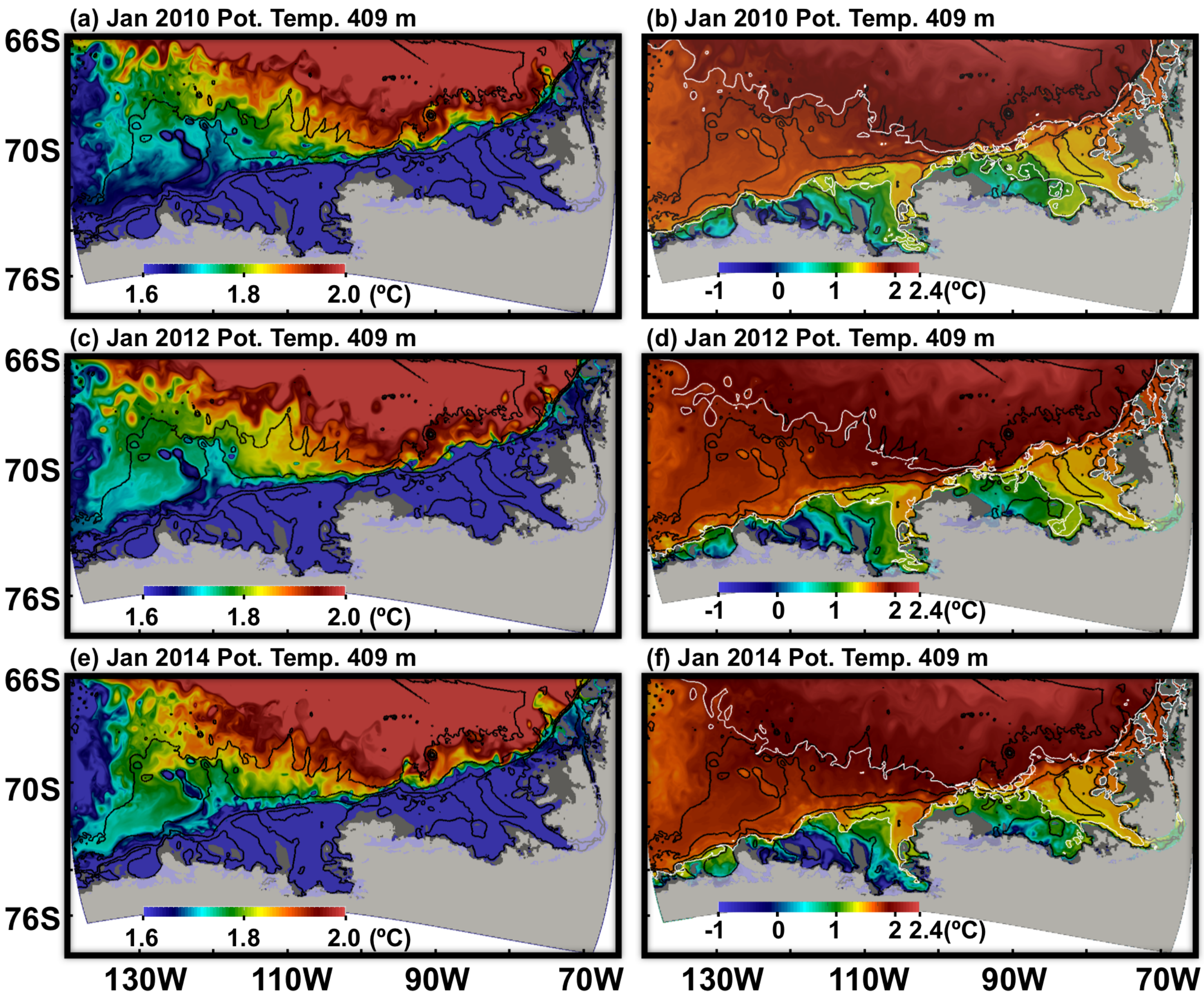

Supplementary Figure 8. Potential temperature at the 409-m depth in January (a, b) 2010, (c, d) 2012, and (e, f) 2014. Note that the same fields are displayed in both the left and right columns but with different color scales in order to emphasize the off- and on-shelf regions, respectively. Isohalines of 34.65 are shown with white lines (b, d, f).

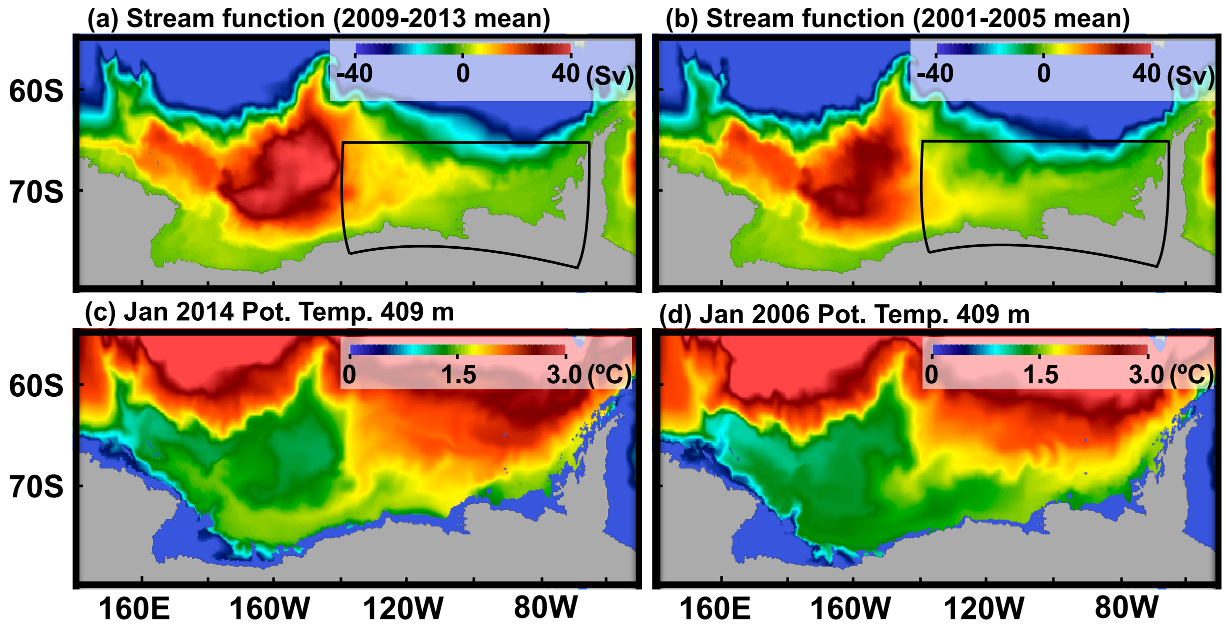

Supplementary Figure 9. Same as Figs. 3a-d but showing the results from ECCO LLC270 global optimization. The regions surrounded by black lines in (a) and (b) denote the regional model domain.

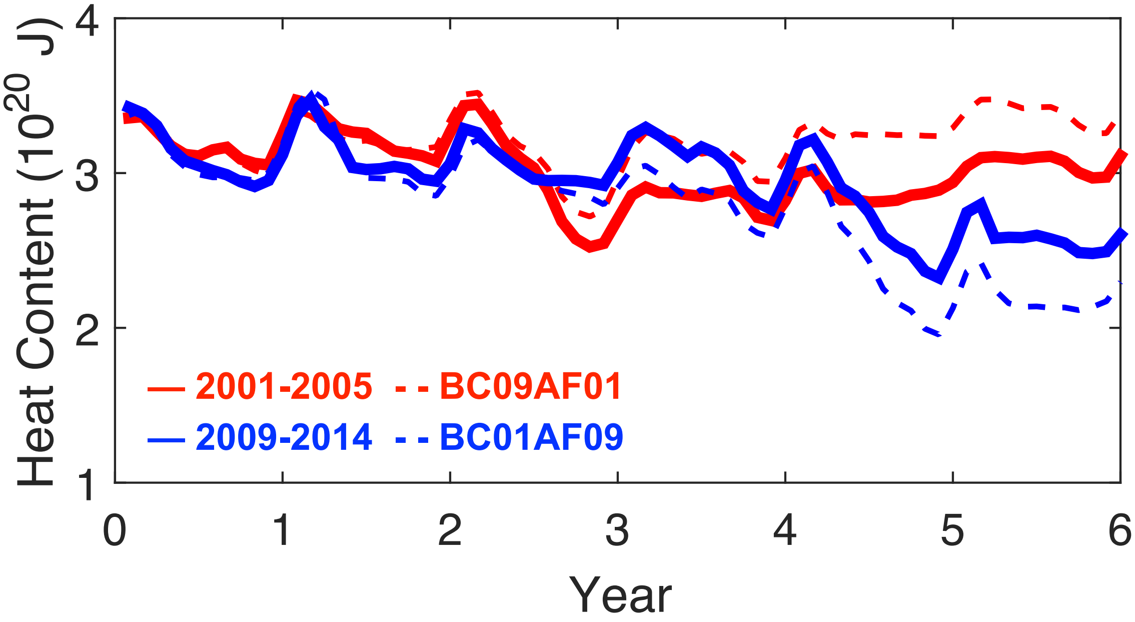

Supplementary Figure 10. Time series of vertically integrated heat content for the eastern AS region (enclosed by the gray lines in Fig. 1) for CTRL(2009-2014) (blue), 2001-2006 (red), BC01AF09 (blue dashed), and BC09AF01 (red dashed) cases. X-axes show years from the start of simulations.

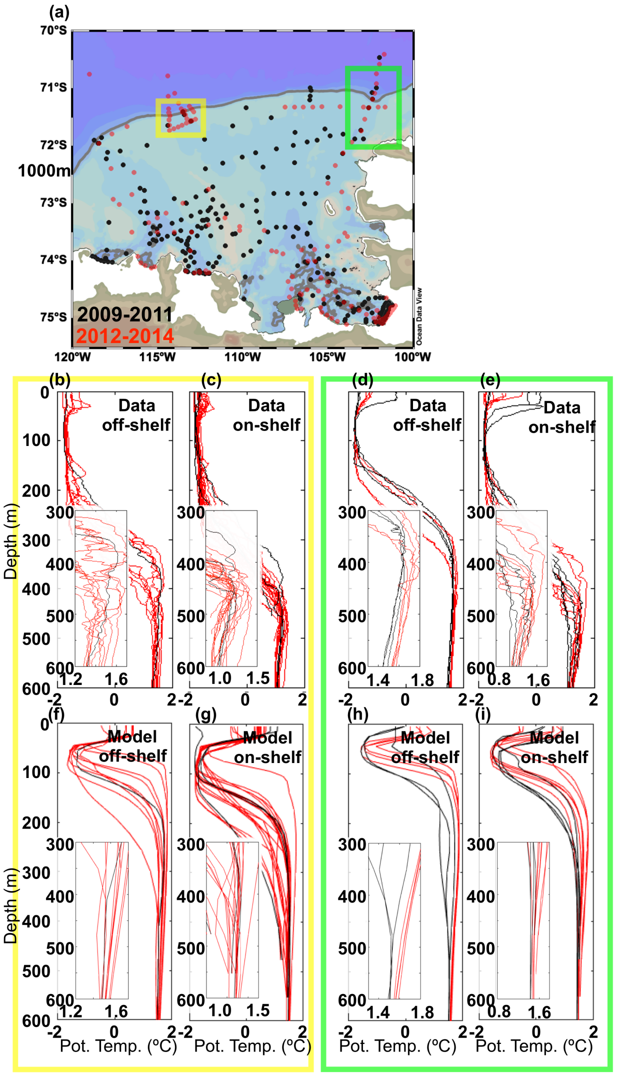

Supplementary Figure 11. (a) Available CTD stations (*3,19-23*) between 2009-2011 (black) and 2012-2014 (red) in the AS. Observed vertical profiles of potential temperature (b) off-shelf and (c) on-shelf near the central trough in the yellow box in (a) and (d) off-shelf and (e) on-shelf near the eastern trough in the green box in (a). (f-i) Same as in (b-e) but displaying simulated vertical profiles of potential temperature. The simulated profiles are sampled using daily output from the nearest locations and times to the observed profiles. For panels (b)-(i), the insets magnify the bottom part of the profiles. Off-shelf is defined as the region with water-depth deeper than 1000m, the gray contour line in (a).

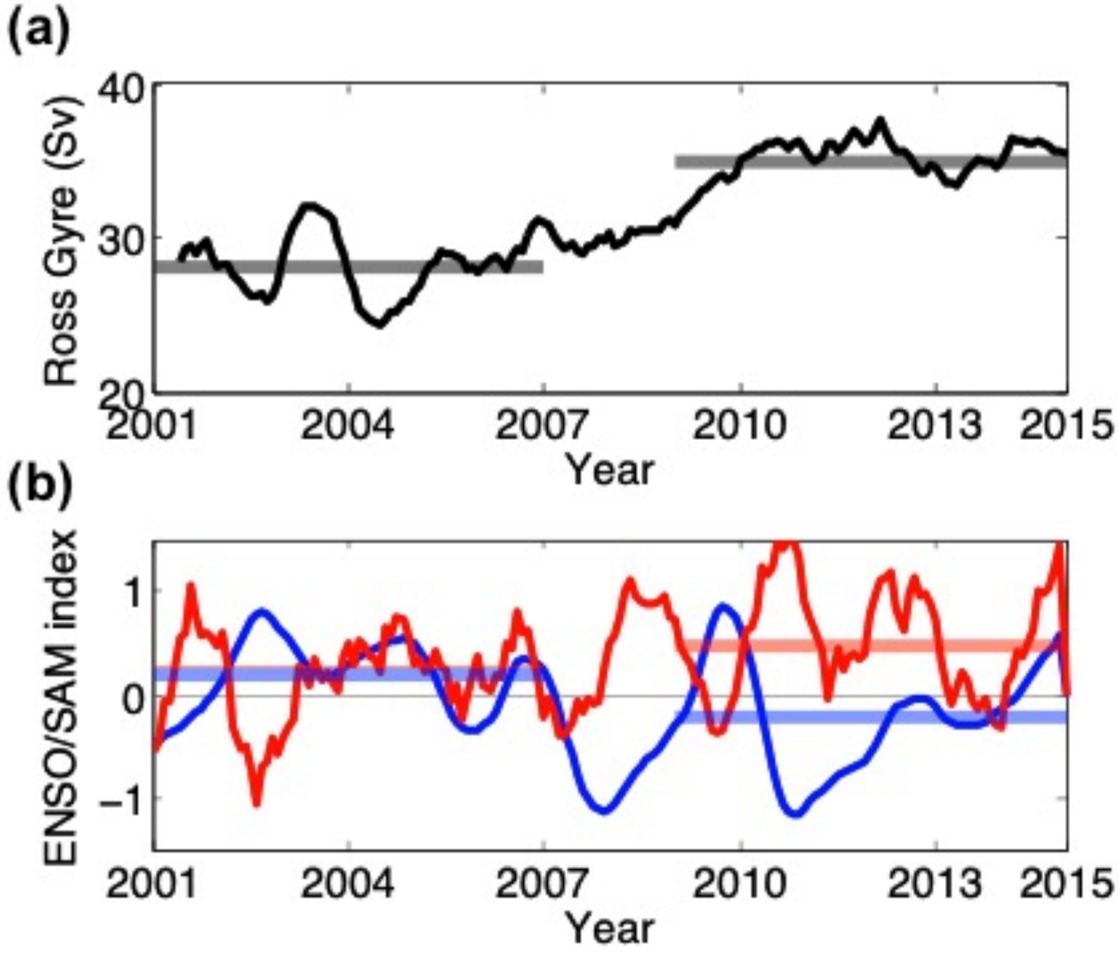

Supplementary Figure 12. (a) Time series of 12-month-running-mean Ross Gyre strength from the ECCO LLC270 global optimization. Ross Gyre strength is defined as the maximum stream function in the Ross Sea. Two gray horizontal bars indicate 2001-2006 and 2009-2014 temporal averages. (b) Time series of 12-month-running-mean SAM (red) and ENSO indices (blue). Similar to (a), two red and blue horizontal bars indicate the time-averaged SAM and ENSO indices, respectively. Note that the red and blue horizontal bars overlap each other for the 2001-2006 period.

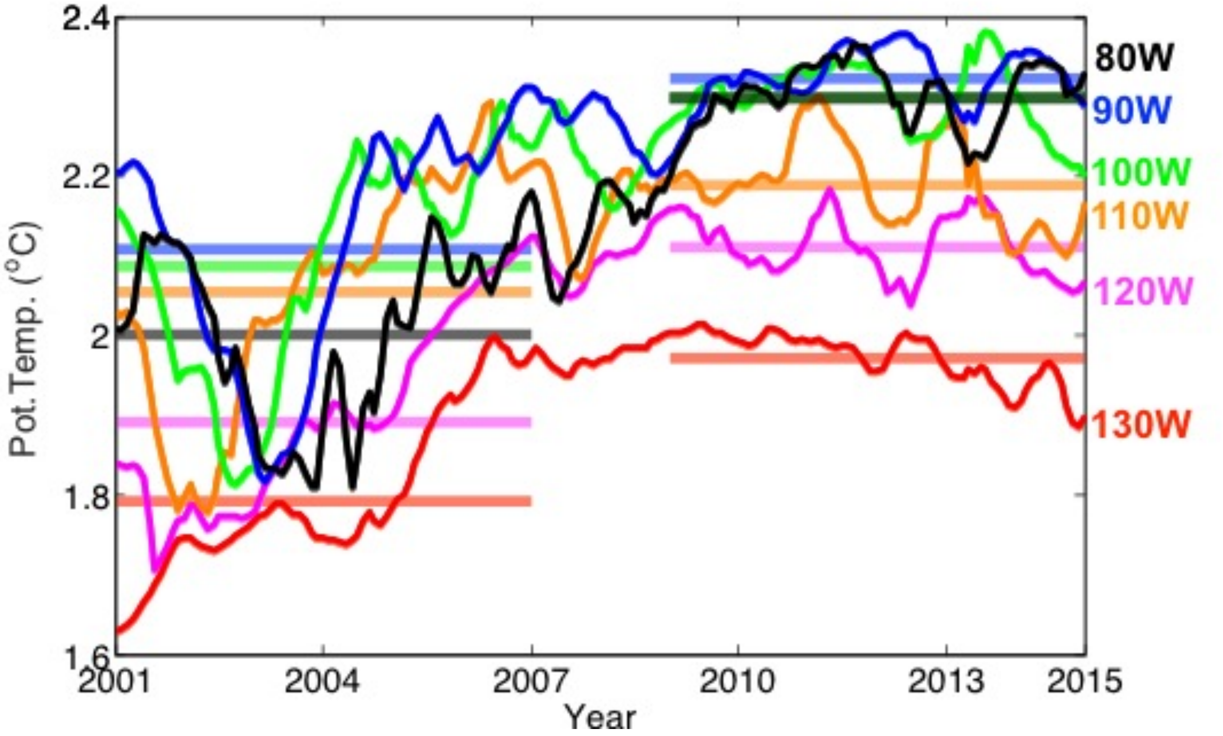

Supplementary Figure 13. Time series of northern boundary 409-m potential temperature at 130W (red), 120W (pink), 110W (orange), 100W (green), 90W (blue), and 80W (black). Horizontal bars are also included to indicate the 2001-2006 and 2009-2014 temporal averages.

Supplementary Table 1. Model parameters used for simulations in this study. Parameters that are different from (*16*) are only shown. Vertical diffusivity is applied for potential temperature, salinity, and other tracer fields.

Parameter	
Background vertical diffusivity (m^2 s^{-1})	5.5 ×10^{-6}
Ocean/air drag coefficient scaling factor	1.0
Air/sea ice drag coefficient	2.0 ×10^{-3}
Sea ice salt concentration	4.0
Stanton number (stable)	0.0327
Stanton number (unstable)	0.0180
Dalton number	0.0346
Lead closing (m)	0.5
Ice strength (N m^{-2})	1.5 ×10^4
Sea ice dry albedo	0.71
Sea ice wet albedo	0.63
Snow dry albedo	0.80
Snow wet albedo	0.65

Supplementary Table 2. Description of sensitivity experiments.

Simulation	Atmospheric forcing	Lateral Boundary conditions
CTRL(2009-2014)	2009-2014	2009-2014
2001-2006	2001-2006	2001-2006
BC01AF09	2009-2014	2001-2006
BC09AF01	2001-2006	2009-2014

Supplementary Table 3. Satellite-based estimates of basal melt rate (*5*) and model mean basal melt rates for 2009-2014 and 2001-2006 for West Antarctic Ice Shelves. The values of heat transfer coefficient γ_T used for our simulations are shown and can be compared to the initial value of 1.0×10^{-4} m s^{-1}. Acronyms of ice shelves used in the figures are shown in brackets.

Name	γ_T ($\times 10^{-4}$ m s^{-1})	CTRL (2009-2014) (Gt yr^{-1})	2001-2006 (Gt yr^{-1})	Observational based estimates from (*5*) (Gt yr^{-1})
George VI (Geo)	0.0935	**88**	**98**	**89.0 ± 17**
Wilkins (Wi)	0.210	**22**	**34**	**18.4 ± 17**
Bach (Ba)	0.364	**13**	**17**	**10.4 ± 1**
Stange (St)	0.0935	**5**	**12**	**28.0 ± 6**
Ferrigno (Fe)	2.81	**4**	**6**	**5.1 ± 2**
Venable (Ve)	0.355	**15**	**20**	**19.4 ± 2**
Abbot (Ab)	0.220	**47**	**60**	**51.8 ± 19**
Cosgrove (Co)	0.253	**7**	**9**	**8.5 ± 2**
Pine Island (Pi)	0.541	**92**	**104**	**101.2 ± 8**
Thwaites (Th)	1.32	**94**	**108**	**97.5 ± 7**
Crosson (Cr)	0.870	**23**	**27**	**38.5 ± 4**
Dotson (Do)	0.558	**37**	**42**	**45.2 ± 14**
Getz (Get)	0.435	**111**	**148**	**144.9 ±14**
