# Supplementary material for: Origin of Circumpolar Deep Water intruding onto the Amundsen and Bellingshausen Sea continental shelves
Source: Nat Commun. 2018 Aug 24;9:3403. doi: 10.1038/s41467-018-05813-1 (PMC6109117; doi:10.1038/s41467-018-05813-1)
Supplement: Supplementary file 3 — Description of Additional Supplementary Files [file 41467_2018_5813_MOESM3_ESM.pdf]

Description of Additional Supplementary Files

File Name: Supplementary Movie 1
Description: Monthly mean spatial distributions of vertically integrated tracers (TR3). Monthly mean spatial distributions of vertically integrated tracers (TR3) representing CDW from 2009 (January) to 2014 (January).
